# Clinics

**Published:** 1902-09-15

**Authors:** 


### CLINICS.

At the Michigan State Dental Association, June 11, 1902.

*Instrument Sharpener.* Dr. B. Bannister, of Kalamazoo, Michigan, presented for his clinic a device for holding dental instruments at definite angles when honing them. It consisted of a small steel block on the side of which was attached an arrangement by which excavators could be held at any angle desirable by means of a small adjusting screw. This steel block is pushed over the hone, oiling at the same time by means of an oil pad attached to the block. By means of this little device any instuments can be sharpened to any shape or at any angle desired.

*Repairing Vulcanite Plates.* Dr. Achillis, of Muskegon, Michigan, gave a clinic on method of repairing rubber plates. The Doctor had a solution with which, if the plate had a mere fracture, he could unite the two pieces together and vulcanize without the packing of any rubber. If it were a case of a broken tooth, the fractured tooth should be removed from its place, being careful not to destroy the labial or buccal aspect. After grinding the tooth to place, cut out the rubber on the lingual side and apply the solution and then pack with rubber, the tooth having been previously secured in place, it is then invested in the flask and vulcanized without separation. The Clinician maintained that all simple fractures could be repaired by simply uniting together with this solution and vulcanizing, and that it was not necessary to cut out and pack in with new rubber.

*Dry Investment.* Dr. Myers, of Saginaw, Michigan, presented a method of soldering in a dry investment. The tooth is ground to fit, then backed and prepared for soldering. The borax is then applied to the backing and it is allowed to dry, and the tooth is then buried up to the edge of the backing in the ashes of the anthracite coal and soldered.

*Metal Crowns.* Dr. G. M. Brown, of Muskegon, Michigan, presented a method for making gold crowns for anterior teeth. His outfit consist of a set of nine steel dies by which he shapes the buccal facings of the gold crowns. He swages directly into lead and thus avoids getting wrinkles on the labial surfaces. After getting the labial contour he then gets it to the shape desired by the use of contouring plyers, fitting on the back piece to the necessary size to fit root and completes the crown with solder.

*Gasoline Burners.* Dr. R. C. Brophy, of Chicago, presented his gasoline outfit which consisted of a bunsen burner, gasoline blow pipe and porcelain furnace. The bunsen burner consists of a tank containing gasoline which supplies the burner which is attached to the tank by a swivel which allows the flame to be thrown in any direction required. There is a pump attached to the tank of the burner a few strokes of which will run the burner for an hour or more. There is also a device at the side of this burner for the attachment of a blow pipe, the latter however requiring the use of a bellows. An ordinary burner without this side attachment can be obtained.

The porcelain furnace has a fire clay muffle and is large enough to accomodate a six-tooth bridge. If desired the muffle can be obtained with a nickel covering. The Doctor claims this furnace is capable of baking the higher fusing bodies in from four to eight minutes. He claims to get from 2,500° to 2,800° of heat.

*Swaging Device.* Dr. Howell, of Ann Arbor, demonstrated some of the uses to which the Lauderdale swaging device can be adapted. This swaging outfit consists of a round chilled steel tube about four inches long into which a small steel cup will fit which will hold a small quantity of sealing wax; the other portion is a steel plunger ground to fit this tube one end hollowed out slightly to receive a wad of unvulcanized rubber which is used as the swaging material. The plunger is long enough so when it is fitted into tube it extends considerably above end of same so as to allow it to be struck by a swaging hammer.

Three important uses to which it could be applied were demonstrated; first the swaging of a cusp for a natural bite crown; second adapting backings to porcelain facing, using a rubber tooth if desirable; third the making of all gold dummies for posterior bridges.

In the first case after the band is fitted to the root and trimmed to proper height and contoured, a natural bite is taken by means of plaster preferably, and the cusps are then carved as desired. Using this as a die an impression is made in the sealing wax which has been softened by heat in the steel cup; after allowing it to cool and harden a cusp is then swaged into this matrix by means of the plunger holding the vulcanite. The thinner the gold used the more distinct the cusps, this cap can then be flowed evenly full with solder which makes solid and strong cusps.

In backing porcelain teeth, after the tooth is prepared for the place to which it is being adapted, the backing material is then cut to an approximate size and the holes punched and fitted to the back of the tooth. This tooth is then sunken into the sealing wax, back up. The backing is then put in position, and swaged into place. So well will this be swaged on the back of tooth that if a vulcanite tooth is used the number of mold can be distinctly seen through the metal.

In making gold dummies, after the gold crowns for the bridge are completed and mounted on the model or put in position in the mouth, porcelain teeth are selected which most nearly fit the space they are to occupy, an impression is then made of each vulcanite tooth in the sealing wax, and into the matrix so obtained the crown material is swaged, a most perfect dummy being made thereby. It is then flowed full of solder and ready to attach to bridge.

*Demonstration of X-ray, by Dr. Hickey.* Preliminary to my demonstration I would like to say that there has been a great deal of criticism in reference to the X-ray. Two or three years ago at the meeting of the State Medical Society one of the prominent surgeons of this state arose to talk about the X-ray, he did not believe in its use and offered as a reason a skiagraph which has been taken to show a supposed fracture of a surgical neck of humerus but the picture seemingly showed a fracture of the clavicle. The Doctor misled by the picture could find no fracture of the clavicle so he claimed the skiagraph was wrong. But further developments proved that it was the case of a young boy in which complete ossification hat not taken place, and what appeared to be a fracture in a picture was simply a cartilaginous portion of clavicle. This criticism showed he did not appreciate the conditions under which the skiagraph was made. And many criticisms are made upon such insufficient basis. I will now proceed with my demonstration.

The induction coil which I have here is a twelve-inch coil and this is a Wehnelt interrupter. In this kind of work it is necessary that the current should be of very high voltage and of very little ampere strength for this reason the induction coil is a very large secondary as compared with the primary. It is also very necessary that there should be perfect insulation between the primary and secondary coil and between the secondary wires themselves. The current generated by this coil passes into the tube at the anode jumps the space and is reflected off the cathode, which is this little platinum target. The X-ray itself is invisible, what you see in the tube is the fluorescence.

The film which is used to take these pictures is regular kodak film which is wrapped in black paper. In placing this film in the mouth it must be perpendicular to the direction of the maximum radius of the ray. (A patient was then presented with a missing cuspid tooth.) It is necessary of course that the patient should be kept perfectly quiet while a radiograph is being taken, he should be about eighteen or twenty inches from the tube, any nearer or farther would make the picture much distorted. The film should be adapted as closely as possible to the part taken.

The treatment of this films after exposure is much the same as that of a common photograph film, except it is necessary in the development to carry it much farther than ordinary photograph negatives. This is the mistake the photographers invariably make when you take them your first films, they think they will require about the same length of time to develop as an ordinary negative, but the development of an X-ray film must be carried to a point which would utterly ruin a photograph negative.

Work on the antrum depends upon the shape of the maxilary arch, if it is such that you can get the film well up into the mouth you can obtain a very good picture which will detect any foreign substance in the antrum, the fluid will also be detected because it is quite opaque to the X-ray. The X-ray is often used to ascertain the height of the fluid in the antrum, if the antrum is gorged with pus it can be readily detected. In reference to the life of an X-ray tube it has not been definitely ascertained what it is that determines its longevity. Out of ten tubes made by the same manufacturer probably not one would resemble the other. It is possible by sending too strong a current through the tube to puncture it. This tube which I have here can be regulated by means of a little rubber tube on the side. If the vacuum becomes too high the screw will raise the rubber valve and let a small amount of air into this capillary tube and reduce it to its proper vacuum.

*Q.:* If a physician has an X-ray apparatus for general use what addition will he be compelled to make for dental purposes?

*A.:* You will require nothing special except a tube of lower vacuum because the degree of penetration in your work is less than that required by the physician usually.

